# Clinical associations between gout and multiple sclerosis, Parkinson’s disease and motor neuron disease: record-linkage studies

**DOI:** 10.1186/s12883-015-0273-9

**Published:** 2015-02-28

**Authors:** Julia Pakpoor, Olena O Seminog, Sreeram V Ramagopalan, Michael J Goldacre

**Affiliations:** Unit of Health-Care Epidemiology, Nuffield Department of Population Health, University of Oxford, Oxford, OX3 7LF UK; Department of Physiology, Anatomy and Genetics and Medical Research Council Functional Genomics Unit, University of Oxford, Oxford, UK

**Keywords:** Multiple sclerosis, Parkinson’s disease, Motor neuron disease, Uric acid, Gout

## Abstract

**Background:**

Uric acid has antioxidant effects on neurons. Abnormally high levels of uric acid are, however, associated with gout. Previous studies have suggested that high levels of uric acid (and the presence of gout) may exert a protective effect against the risk of developing some neurological diseases. We aimed to investigate this hypothesis in a large database of hospital admissions in England.

**Methods:**

We analysed a database of linked statistical records of hospital admissions and death registrations in England (1999–2012). A cohort of people with gout was constructed and followed for development of multiple sclerosis (MS), Parkinson’s disease (PD) or motor neuron disease (MND). Then, conversely, cohorts of all people in the database with MS, PD or MND were constructed and followed for subsequent gout. Rate ratios (RRs) were determined, comparing these cohorts with people in a reference cohort.

**Results:**

In the gout cohort, we observed a modest elevation of the overall risk of subsequent MS, PD and MND (respectively, RR = 1.27 (95% confidence interval 1.03-1.55), 1.11 (1.05-1.17) and 1.28 (1.11-1.48) which was largely attributable to an increased risk observed in the early years after hospitalisation for gout. The increased risk of neurological disease did not remain after 5 years. In the cohorts of people with MS or PD, there was a significantly reduced risk of subsequent gout admission (RR = 0.79 (0.69-0.89) and 0.83 (0.79-0.87), respectively). This inverse association was sustained over time. There was also a reduced risk of MND following gout which only emerged more than five years following initial gout admission (RR at 5+ years 0.35 (0.15-0.68)).

**Conclusions:**

This study investigated the epidemiological evidence for a protective role of high serum concentration of uric acid, for which we used gout as a proxy, in the aetiology of MS, PD or MND. Our observations do not support this hypothesis. However, when the order was reversed, and we retrospectively followed up patients with MS, PD and MND for a number of years, we found a statistically significant deficit of gout. This suggests that there is relationship between some aspects of these neurodegenerative diseases and metabolism of uric acid.

## Background

Oxidative stress is thought to have an important role in neuronal degeneration. Neuronal degeneration is a characteristic feature of several neurological disorders including multiple sclerosis (MS), Parkinson’s disease (PD), and motor neuron disease (MND) [1,2]. A high fatty acid content and a low antioxidant capacity are thought to be factors which render neural cells more susceptible than other body tissues to oxidative stress. This, in turn, may result in damage to proteins and DNA, subsequent inflammation and neuronal loss [3-5]. Uric acid is a potent antioxidant found throughout extracellular fluid, as sodium urate, and is thought to account for more than half of the antioxidant capacity of plasma [6]. Levels of serum urate are closely correlated with levels of urate in the cerebrospinal fluid (CSF) and CSF urate is typically 10% of peripheral levels. This suggests that CSF levels of urate are in part dependent on serum uric acid levels [7]. It has been suggested that uric acid may have neuroprotective influence as a scavenger of reactive nitrogen and oxygen radicals. A powerful antioxidant effect of uric acid on neurons has been demonstrated through in vivo and in vitro studies [8,9]. The hypothesis that high levels of uric acid exert a protective effect against oxidative neuronal damage is supported by studies showing that individuals with PD, MS and MND have significantly lower serum uric acid levels than healthy controls [10-12]; and there is some evidence that low uric acid levels correlate with progression of symptoms and disease relapse [13,14]. Among men participating in a 30-year prospective study, men with elevated uric acid levels had a 40% reduction in the incidence of PD, unaffected by adjustment for age and smoking [15]. Similarly, a large nested case–control study found that higher plasma concentrations of urate predict risk of PD, with a significantly reduced rate of PD for men in the highest quartile of uricemia compared with the lowest [16]. This notion is strengthened by the finding that a diet which increases plasma urate levels is associated with a reduced PD risk, rendering it unlikely that the association is merely a consequence of shared predisposing factors [17].

Abnormally high levels of serum uric acid are, however, known to be pathological and most commonly associated with gout, a form of recurrent acute inflammatory arthritis in which uric acid crystallizes and is deposited within joints [18]. Individuals with gout, and therefore high levels of serum uric acid, have been postulated to have greater protection from neurodegenerative diseases in which oxidative stress is thought to contribute to pathogenesis. A lower risk of PD and MS in individuals with gout has been previously reported [19-21]. The relationship between gout (as an indicator of high levels of uric acid) and the incidence of neurodegenerative diseases is of potential therapeutic interest given the proposed possibility that elevation of uric acid levels may reduce the incidence of neurological disease. However, it is currently unclear if lower levels of uric acid in individuals with MS, PD and MND represent a cause or consequence of the disease. This record linkage study therefore aimed to extend previous findings by determining the risk of PD, MS and MND in individuals with a history of gout, and, conversely, the risk of subsequent gout in individuals with PD, MS or MND.

## Methods

### Population and data

We used a linked English national data set of hospital admissions (Hospital Episode Statistics (HES) and mortality. HES data are records of hospital care that are compiled for every episode of hospital day case care (when the patient is admitted to hospital but does not stay overnight) or hospital inpatient care in all English National Health Service (NHS) hospitals, and they were supplied by the English national Information Centre for Health and Social Care. The mortality data, derived from death registrations, were supplied by the Office for National Statistics. The linked dataset used in this study, in which successive records for each individual were linked together, was constructed by the Oxford record linkage group. The International Classification of Disease (ICD) codes used for gout were 274 in the ninth revision and M10 in the tenth. The ICD 9 and 10 revision codes used for MS, PD, and MND were, respectively, 340 and G35, 332.0 and G20, and 335.2 and G12.2.

### Gout before MS, PD, or MND

The methods of analysis were the same for all neurological diseases; we describe the methods for gout followed by MS as the example. A cohort of people with gout (the ‘exposure’ cohort) was constructed for people with a diagnosis of gout in an episode of hospital care by identifying the first episode of day case care, or admission, for gout during the study period. A reference cohort was constructed by identifying the first admission for each individual with various other mainly minor medical and surgical conditions as described in previous studies of disease associations [22]. Standard epidemiological practice was followed by selecting a diverse range of conditions rather than relying on a limited range (in case the latter are themselves atypical in their risk of neurological disease). As a check, we estimated the risk of each neurological disease in the reference cohort to ensure that it does not include conditions that have atypically high or low rates of neurological disease.

People were included in the gout or reference cohort if they did not have an admission for MS either before or at the same time as the admission for gout or the reference condition. The database was then investigated for any subsequent NHS hospital care for, or death from, MS in these cohorts (the ‘outcome’ condition). We considered that rates of MS in the reference cohort would approximate those in the general population while allowing for migration (data on migration by individuals covered by the datasets was not available).

### Statistical methods

Rates of MS were calculated based on person-days. Date of entry into each cohort was the date of first admission for gout, or reference condition, from the start of data collection on 1^st^ January1999 and date of exit was the date of first record of MS, death, or the end of data collection (31^st^ March 2012), whichever was the earliest. We calculated rates for MS, stratified and then standardised by age (in five-year age groups), sex, calendar year of first recorded admission, region of residence, and quintile of patients’ Index of Deprivation score (a standard English measure of socio-economic status). The indirect method of standardisation was used, with the combined gout and reference cohorts as the standard population. We applied the stratum–specific rates (e.g. within each five-year age stratum) in the standard population to the number of people in each stratum (e.g. within the same five-year age stratum) in the gout cohort and then, separately, to those in the same stratum in the reference cohort, to obtain the expected number of people with MS in each stratum of the gout and reference cohort. Observed and expected numbers were then summed across all strata to give totals for all strata combined. Rate ratios (RRs) were calculated by taking the standardised rate of occurrence of MS in the gout cohort relative to the reference cohort using the formula (O^gout^ / E^gout^) / (O^ref^ / E^ref^), where O and E are the observed and expected numbers of MS cases in the gout and reference cohorts, respectively. The 95% confidence interval (95% CI) for the rate ratio of MS and χ^2^ statistics for its significance were calculated, with continuity correction for small numbers, as described elsewhere [23].

### MS, PD, and MND before gout

We then used the same methods to study the neurological diseases followed by gout. We constructed ‘exposure’ cohorts of people, separately, comprising all people in the dataset with MS, PD, or MND. We compared them with the reference cohort. In this analysis, we excluded anyone with gout prior to or at the same time as their first admission for neurological disease or reference cohort condition. Taking both study designs – gout followed by neurological disease, and neurological disease followed by gout – the exclusions ensured that no individual was double-counted as a case of gout both before and after neurological disease.

The datasets are in the custodianship of the Unit of Health-Care Epidemiology and are not freely available. Ethical approval to construct, maintain, develop and analyse the datasets has been obtained, on an ongoing basis, from the Central and South Bristol Multi–Centre Research Ethics Committee (04/Q2006/176).

## Results

### Gout before MS, PD or MND

Basic demographic information of the disease cohorts is shown in Table 1. The number of people admitted to hospital with gout was 214,653. There were approximately 9 million people in the reference cohort. Comparing the gout cohort with the reference cohort, the rate ratios (RR) for subsequent MS, PD and MND were, respectively, 1.27 (95% confidence interval 1.03-1.55), 1.11 (1.05-1.17) and 1.28 (1.11-1.48), Table 2. Results were no longer significant upon inclusion only of cases of neurological disease which occurred at least five years following initial gout admission (Table 2).

**Table 1:** **Demographic characteristics of disease cohorts**

**Age (years)**	**Gout**		**Multiple sclerosis**		**Parkinson’s disease**		**Motor neuron disease**	
	**Age (%)**	**Male (%)**	**Age (%)**	**Male (%)**	**Age (%)**	**Male (%)**	**Age (%)**	**Male (%)**
<25	0.1	76.7	2.1	30.4	0	53.7	1.6	64.0
25-44	4.9	92.4	29.1	29.3	0.5	57.8	4.1	64.9
45-54	9.0	91.1	25.6	29.7	1.5	61.9	8.0	63.0
55-64	15.7	86.9	21.9	33.7	6.2	63.7	19.3	59.9
65-74	23.9	78.5	13.3	32.6	22.8	61.3	31.6	56.8
75+	46.3	61.4	8.1	28.2	68.9	53.6	35.4	53.0
Total	100	73.7	100	30.8	100	56.2	100	57.0

**Table 2:** **Occurrence of multiple sclerosis (MS), Parkinson’s disease (PD) and motor neuron disease (MND) in patients hospitalised with gout by time interval: observed (O) and expected (E) number of cases in each cohort, relative risk (RR) with 95**% **confidence intervals (95**% **CI) in the exposure cohort compared with the reference cohort** ^**a**^

**Time interval**	**Gout before MS**			**Gout before PD**			**Gout before MND**		
	**O**	**E**	**RR (95% CI)**	**O**	**E**	**RR (95% CI)**	**O**	**E**	**RR (95% CI)**
Overall	98	77.3	1.27 (1.03-1.55)	1568	1420.3	1.11 (1.05-1.17)	197	155.1	1.28 (1.11-1.48)
<1 yr	36	23.5	1.55 (1.08-2.15)	405	330.9	1.24 (1.12-1.38)	59	39.0	1.56 (1.17-2.04)
1-4 yrs	39	36.9	1.06 (0.75-1.45)	824	745.8	1.11 (1.03-1.19)	95	80.4	1.19 (0.96-1.46)
5+ yrs	23	16.8	1.37 (0.87-2.06)	339	343.6	0.99 (0.88-1.10)	43	35.6	1.21 (0.87-1.64)

^a^Conditions used in reference cohort, with Office of Population, Censuses and Surveys (OPCS) code edition 4 for operations and ICD10 code for diagnosis (with equivalent codes used for other coding editions): adenoidectomy (OPCS4 E20), appendectomy (H01–H03), dilation and curettage (Q10–Q11), hip replacement (W37–W39), knee replacement (W40– W42), cataract (H25), otitis (H60-H67), upper respiratory tract infections (J00-J06), varicose veins (I83), haemorrhoids (I84), deflected septum (J34.2), nasal polyp (J33), impacted tooth and other disorders of teeth (K00–K03), inguinal hernia (K40), in-growing nail, toenail and other diseases of nails (L60), bunion (M20.1), internal derangement of knee (M23), selected limb fractures (S42, S52, S62, S82, S92), contraceptive management (Z30).

Note that, in analysis, we included all people eligible to be in the reference cohort in each stratum and calculated the observed and expected number of people within each age stratum (see Method). Then, given that the individual stratum-specific values of observed and expected cases were equivalent in respect of age, we summed the age-specific values to provide an age-standardised all-ages set of RRs.

There were 82,220, 217,179 and 25,185 people, respectively, in the MS, PD and MND cohorts. In the cohorts of people with MS, PD and MND, the RRs for subsequent gout were, respectively, 0.79 (0.69-0.89), 0.83 (0.79-0.87) and 0.94 (0.75-1.16), Table 3. Notably, a reduced risk of MS and PD following gout was significant after the first year following admission for gout, and was then sustained long-term. For example, after 5 years following hospitalisation for MS the rate ratio for gout was 0.70 (0.57-0.85) and after hospitalisation for PD 0.52 (0.45-0.59). The risk for gout was not different between the reference cohort and people hospitalised with MND within five years after hospitalisation for MND, but it was significantly and strikingly low at 5 years and more after hospitalisation (RR 0.35, 0.15-0.68), Table 3.

**Table 3:** **Occurrence of gout in patients hospitalised with multiple sclerosis (MS), Parkinson’s disease (PD) and motor neuron disease (MND) by time interval: observed (O) and expected (E) number of cases in each cohort, relative risk (RR) with 95**% **confidence intervals (95**% **CI) in the exposure cohort compared with the reference cohort**

	**MS before gout**			**PD before gout**			**MND before gout**		
**Time interval**	**O**	**E**	**RR (95% CI)**	**O**	**E**	**RR (95% CI)**	**O**	**E**	**RR (95% CI)**
Overall	246	311.7	0.79 (0.69-0.89)	1521	1825	0.83 (0.79-0.87)	83	88.4	0.94 (0.75-1.16)
<1 yr	50	43.1	1.16 (0.86-1.53)	522	489	1.07 (0.98-1.17)	41	29.9	1.38 (0.99-1.87)
1-4 yrs	96	126.4	0.76 (0.61-0.93)	728	893.2	0.85 (0.79-0.92)	34	35.5	0.96 (0.66-1.34)
5+ yrs	100	142.2	0.70 (0.57-0.85)	231	442.8	0.52 (0.45-0.59)	8	23.0	0.35 (0.15-0.68)

See footnotes Table 2.

## Discussion

In the gout cohort, we demonstrated a statistically significant but modest elevation of risk of admission for subsequent MS, PD and MND. After excluding cases of MS, PD or MND observed in the first year after hospitalisation with gout, there was no elevated RR for these conditions. These results do not confirm findings from previous studies suggesting a reduced risk of neurological disease following gout as a consequence of a neuroprotective antioxidant effect of uric acid, or the corollary that individuals with low levels of uric acid have an impaired ability to counteract free radical toxicity [19,20]. It is possible that the inflammatory state associated with the development of symptomatic gout or recurrent inflammatory arthritis (rather than simply hyperuricemia in previous work) counteracts any potential protective antioxidant effect of uric acid. The observed slightly increased risk in early years might be a reflection of surveillance bias, whereby an individual seen in hospital for one condition is more likely to have a second detected. Another possible explanation for the small increase in risk in the early years, that was not sustained long-term, is that individuals admitted to hospital with gout may undertake measures successfully to lower their levels of uric acid (and reduce future gout attacks), which may involve both dietary changes and the use of uric acid reducing medication [24]. Further investigation of the comparison of risk of MS, PD or MND in treated vs untreated hyperuricemia would have enabled better characterisation of the association, but such data was not available in this study.

Further, from hospital admission statistical records alone, we cannot be sure that, in the gout cohort, the onset of gout really did invariably precede the onset of MS, PD and MND. The date of first admission for a neurodegenerative disease is likely to have been preceded by a period of sub-clinical/undiagnosed disease, and thus, the disease state itself may have resulted in somewhat elevated levels of uric acid and an increased risk of gout. The fact that the raised risk was not sustained over time means that we cannot be confident that the findings support a causal role for uric acid in altering neurological disease risk. It needs further investigation to establish whether uric acid levels alter neurological disease risk (and/or disease progression), given the potential therapeutic approach of altering uric acid levels (through either dietary or pharmacological measures) in altering risk of neurological diseases.

In the MS, PD and MND cohorts, we found a prolonged reduced risk of subsequent admission for gout. Notably, for the MS and PD cohorts a reduced risk of gout was seen following the first year of initial admission for neurological disease. In the MND cohort, this reduced risk only appeared five years following initial MND admission. Although, we can only speculate about pathophysiological mechanisms, our findings on gout before and after the neurological diseases might indicate that the causal pathway is that the neurological disease states result in lowered uric acid levels. For example, central nervous system inflammation which is a typical feature of MS has been associated with increased production of the strong oxidant peroxynitrite and free radicals, which may in turn lead to reduced levels of uric acid, which is a peroxynitrite scavenger [25]. A cross-sectional study which found markedly reduced levels of serum urate in MND patients with bulbar onset, and with a longer duration of disease, suggested that the reduced levels of serum urate may be a consequence of malnutrition secondary to the impact of MND, particularly in its later stages [26].

This study’s strengths include its large size and the fact that it is a national study and therefore generalizable as the cohorts are likely to relatively accurately reflect the national prevalence of these neurological disorders. Further, it demonstrates what can be done with the analysis of readily available electronic medical records.

The study also has limitations. Importantly, it is not a cohort study with follow-up from the date of “first ever” diagnosis, but just from “first recorded” diagnosis in a hospital day case or inpatient record. Data are not available on patients who migrate out of England. The dataset is limited to people who were admitted to hospital, or who received day case specialist care, and thus there exists the potential for selection bias in omitting milder cases of gout. Further, given that only a minority of individuals with elevated uric acid levels develop gout, using gout as a proxy for high uric acid levels may underestimate the association between uric acid and neurological diseases. Uric acid levels appear positively correlated with risk of gout, and the gout cohort is therefore more likely to capture those with more severe uric acid elevation in which, over time, urate crystals have deposited in or around joints. There is very limited information on potential confounding factors such as detailed socioeconomic characteristics, beyond the IMD index, ethnicity and smoking.

Studies of alternative design would be costly and complex undertakings. For example, if funds allowed, “shoe-leather” studies of individual patients could be undertaken by personal follow-up, such as following large numbers of people with gout to await development of these three uncommon neurological diseases, or studies could be undertaken of people with MS, PD or MND to identify clinical gout or to measure their serum uric acid levels. The latter would need to be designed in ways that allow the investigators to determine the time sequence of ‘high levels of uric acid before the neurological condition’ or ‘neurological condition before changes in uric acid levels’.

## Conclusions

In summary, the data in our study provide good evidence of an inverse association between gout and MS PD, and MND. Our data therefore supports the notion that the pre-existence of the neurological diseases reduces the occurrence of gout. We highlight the need for future studies to incorporate duration of disease, and effects of treatment, with longitudinal data on uric acid levels to determine whether altered uric acid levels, associated with neurodegenerative disease, are truly a cause or a consequence of neurodegenerative disease.
